# Partnering with women collectives for delivering essential women’s nutrition interventions in tribal areas of eastern India: a scoping study

**DOI:** 10.1186/s41043-017-0099-8

**Published:** 2017-05-22

**Authors:** Vani Sethi, Arti Bhanot, Surbhi Bhalla, Sourav Bhattacharjee, Abner Daniel, Deepika Mehrish Sharma, Rajkumar Gope, Saba Mebrahtu

**Affiliations:** 1 Child Development and Nutrition Section, UNICEF India Country Office, 73 Lodhi Estate, New Delhi, 110003 India; 2 Independent Consultant, New Delhi, India; 3 UNICEF Field Office of Odisha, Bhubneswar, India; 4 UNICEF Field Office of Chattisgarh, Raipur, India; 5 UNICEF Office of Madhya Pradesh, Bhopal, India; 6 Ekjut, West Singhbhum, Jharkhand, India; 7 Independent Consultant, Melbourne, Australia

**Keywords:** Women collectives, Women’s nutrition, Capacity assessment, Grants, Governance

## Abstract

**Background:**

We examined the feasibility of engaging women collectives in delivering a package of women’s nutrition messages/services as a funded stakeholder in three tribal-dominated districts of Odisha, Jharkhand and Chhattisgarh States, in eastern India. These districts have high prevalence of child stunting and poor government service outreach.

**Methods:**

Conducted between July 2014 and March 2015, an exploratory mix-methods design was adopted (review of coverage data and government reports, field interviews and focus group discussion with multiple stakeholders and intended communities) to assess coverage of women’s nutrition services. A capacity assessment tool was developed to map all types of community collectives and assess their awareness, institutional and programme capacity as a funded stakeholder for delivering women’s nutrition services/behaviour promotion.

**Results:**

Limited targeting of pre-pregnancy period, delays in first trimester registration of pregnant women, and low micronutrient supplementation supply and awareness issues emerged as key bottlenecks in improving women’s nutrition in these districts. Amongst the 18 different types of community collectives mapped, Self Help Groups (SHGs) and their federations (tier 2 and tier 3), with total membership of over 650,000, emerged as the most promising community collective due to their vast network, governance structure, bank linkage, and regular interface. Nearly 400,000 (or 20% of women) in these districts can be reached through the mapped 31,919 SHGs. SHGs with organisational readiness for receiving and managing grants for income generation and community development activities varied from 41 to 94% across study districts. Stakeholders perceived that SHGs federations managing grants from government and be engaged for nutrition promotion and service delivery and SHG weekly meetings can serve as community interface for discussing/resolving local issues impeding access to services.

**Conclusions:**

Women SHGs (with tier 2 and tier 3) can become direct grantees for strengthening coverage of women’s nutrition interventions in these tribal districts/pockets, provided they are capacitated, supervised and given safe guards against exploitation and violence.

## Background

Stunting or linear growth failure, which results in irreversible damage to physical and cognitive development of children, is the most prevalent form of undernutrition in India and most severe in tribal population [1, 2]. Growth failure, most of which happens in foetal stage and first 24 months after birth, can be prevented by intervening in pre-conception phase of the life cycle in addition to the “1000 days” window period from pregnancy till child is 2 years old [3, 4]. Thus, adequacy of women’s nutrition before, during, and in between pregnancies is critical for preventing child stunting. The World Health Organization has called for global action to reduce the proportion of children who are stunted by 40% by 2025 [5]. There is substantial scientific consensus on determinants, consequences and proven interventions to address child stunting through women-centric approaches, but the challenge is to translate this understanding to effective strategies that reach the most marginalised population [6]. The five essential nutrition interventions for women include both, that directly support nutrition intake (or nutrition-specific, e.g. food supplementation) and those needed to improve the impact of these interventions (or nutrition-sensitive, e.g. access to health services). These interventions are (1) improving the quantity of household food consumed and its nutrient quality, (2) preventing and managing micronutrient deficiencies and anaemia, (3) increasing women’s access to health services and special care for ‘most vulnerable’, (4) increasing women’s access to water and sanitation education and commodities, and (5) preventing too early, too many and too close pregnancies. Community engagement in improving last mile delivery of health and nutrition interventions has been researched and valued, but has largely remained honorary work. Evidence from randomised controlled trials within and outside India suggests that using women groups as platforms for promoting health interventions is a feasible approach in low resource settings, provided requisites such as high quality facilitators for establishing and maintaining the group, high coverage of intervention, sufficient time for implementation of the intervention, concomitant supply strengthening interventions and appropriate safeguards against harms such as conflict with service providers and domestic violence are met [7–11]. Notable global examples where community collectives have been partnered with, in equal capacity as a grantee and fund manager for delivering services and promoting health and nutrition behaviours in underserved communities include Community Conditional Transfer programme in Indonesia, livelihood and food security programmes in Bangladesh (Shouhardo, Jibaon-o-Jibika) and Nepal (Suaahara) [12, 13]. Indian experiences include Kudumbashree (Kerala), Society for Elimination of Rural Poverty Project (Andhra Pradesh and Telangana), Self Employed Women’s Association-rural (various states), Community Health Care Management Initiative (West Bengal), Jamkhed model (Maharashtra) and urban health models by Urban Health Resource Centre and Mahila Abhivrudhi Society, Andhra Pradesh. All these experiences build on bank linkages of women collectives and government or non-government organisation (NGO) as their promoting agency. Women’s groups are trained on promotion of the health and nutrition interventions, the scope and duration of training varying with the type of programme. The promoting agency (government or NGO) provides capacity building and supervisory support. Most programmes strengthen the health services delivery system in addition to intervening with community groups. This study assesses feasibility of community collectives as a funded stakeholder that is; their readiness to receive and manage grants for delivery of essential women’s nutrition interventions in eastern India tribal regions with the highest prevalence of stunting.

## Methods

Geographic scope of the study was three tribal dominated districts—Bastar, Koraput and West Singhbhum in eastern Indian states of Chhattisgarh, Odisha and Jharkhand, respectively. Selection of districts was consultation with the state government given this study findings were envisaged as precursor and guide to a demonstration programme with women collectives in these districts.

The study conducted from July 2014 to March 2015, adopted a mix-methods exploratory study design with two phases an exploratory and capacity assessment). The exploratory phase included a situation assessment of women’s nutrition status and services in the study districts.

For this, existing district level data sources on demography, health and nutrition status, services and behaviours were identified through published literature and review of websites and latest publications of relevant government departments and development agencies. Estimates have been reported from Census 2011, Annual Health Survey (AHS) 2012–13, District Level Household Survey (DLHS) 2 and 3 and HUNGaMA survey. A database of all relevant indicators was prepared to measure coverage of essential nutrition interventions. Data analysis was undertaken on MS Excel.

For capacity building phase, mapping of community collectives was done through a preliminary step of identifying promoting agencies (government or NGO).

A community collective was defined as “any village-based institution, which was organised into a group with a minimum of 8–10 village residents, may or may not be aggregated into a collection of such groups/members as higher tiers; the members being primarily women or in mixed groups with men and holding periodic meetings for specific action oriented functions, may or may not be pertaining to thrift and credit.”

**Fig. 1:**
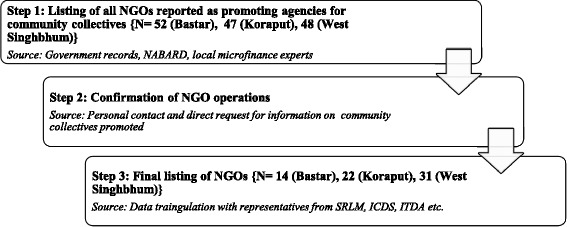
Process for mapping NGOs serving as promoting agencies for community collectives

Number of promoting agencies mapped were 17, 28 and 33 in Bastar, Koraput and West Singhbhum, respectively, of which 14, 22 and 31 were NGOs (Fig. 1). A mapping tool was developed to garner information on types, numbers, purpose and functionality of various community collectives virtually from the identified promoting agencies. Validation of reported information was done through third party discussions with government officials at sub-district level. “Matured” collectives at first tier (operational for atleast 1 year, held regular meeting with 70 to 80% of member attendance, had bank linkage, were involved in income generation activities and were receiving bank credit) and second tier (holding general body meetings with tier 1, supporting tier 1 in thrift and credit activities) were sampled for capacity assessment phase.

**Fig. 2:**
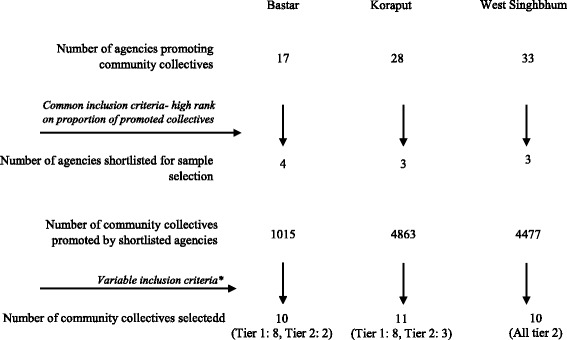
Sampling procedures for selecting community collectives for capacity assessment. *tier 1 and 2 collectives selected separately in Bastar and Koraput, while only collectives federated at tier 2 considered for sampling in West Singhbhum

In the capacity assessment phase, of the 17, 28 and 33 agencies promoting community collectives in Bastar, Koraput and West Singhbhum, 4, 3 and 3 agencies were shortlisted, based on high rankings on proportion of collectives promoted in the district to the total number of collectives. Using the number of collectives promoted by these shortlisted agencies 10, 11 and 10 “matured” collectives were purposively selected in Bastar, Koraput and West Singhbhum through a consultative approach (Fig. 2).

Community demand, facilitators and barriers for women’s health and nutrition enhancement and perspectives on involving community collectives in social issues was assessed through focus group discussions (FGDs) with adolescent girls, reproductive age women and older women and with frontline workers such as accredited social health activist and anganwadi worker. District and block level officials of government agencies like State Rural Livelihood Mission (SRLM), Integrated Tribal Development Agency (ITDA) and representatives from NGOs were also interviewed. Five different sets of capacity assessment tools were developed, field tested and standardised for use across three districts. Two personnel with experience in field research, understanding of the local context and dialects undertook the primary data collection in each district. The interviews and FGDs were transcribed word-by-word. Non-verbal cues and any observations on group dynamics were noted. Data collection and analysis was simultaneous in the assessment phase to have better in-sight for future rounds of interviews and discussions. MS excel was used for coding and summarization of findings.

## Results

The total population ranges from 1.3 to 1.5 million across the study districts. All three districts are rich in natural resources, have dense forest cover and are home to various tribe groups. Agriculture is the main occupation, with reliance on traditional, rain-fed farming techniques. The population is exposed to the known socio-demographic determinants of stunting such as high fertility rates and low adult literacy (Table 1).

**Table 1:** Demographic profile of Bastar, Koraput and West Singhbhum

Indicators	Bastar^a^	Koraput	West Singhbhum
Population (total)	14,13,199	13,79,647	15,02,338
Population (male)	6,98,487	6,78,809	7,49,385
Population (female)	7,14,712	700,838	7,52,953
Child population (0 to 6 years)	2,16,713	2,25,126	2,61,493
Population density (population per sq km)	135	156	208
Decadal growth rate	18.3	16.6	13.5
Sex ratio at birth	930	911	983
SC population (%)	2.7	14.2	3.8
ST population (%)	66.3	50.6	67.3
PVTG (n)	NA	NA	1823
Adult literacy (%)	66.3	49.9	58.6
Households (n)	3,10,359	3,37,677	3,02,046
Household size	4.5	4.1	5
Area (sq km)	4030	8807	7224
Blocks	7	14	18
Villages	611	1985	1792

*Source*: Census of India 2011

^a^Data for undivided district including Kondagaon except area, block and village data

*NA* (No PVTG in that district)

### Nutritional status of women in study districts

As per the Annual Health Survey, about 13% of women in the age group of 18 to 59 years in Bastar, 44% in Koraput and 27% in West Singhbhum had body mass index below 18.5 kg/m^2^. Prevalence of anaemia amongst women in the same age-group was 80% or higher across all three districts. Data on anaemia in pregnancy dated DLHS 2002 according to which all pregnant women were mild to moderately anaemic in the study districts. More were likely to be severely undernourished with the revised cut-offs for classification of anaemia in 2011 than reported in this survey [14].

### Coverage of the five essential nutrition interventions in study districts

Under intervention 1, i.e. improved food and nutrient intake (both in quantity and quality), availability of household food ration through the government’s Public Distribution System (PDS) and fortified supplementary food through Integrated Child Development Services (ICDS) were ascertained. PDS coverage ranges from 45% in Koraput to 81% in West Singhbhum (Table 2). Non-availability of ration cards, an income certification proof, was cited as a reason for inability to avail PDS services in all study districts. Anganwadi Centres, or the village platform that delivers services under ICDS, exist in all villages in Koraput and West Singhbhum. However, irregularity of food distribution and the poor quality of supplementary food are common.

**Table 2:** Coverage of five essential interventions across the three study districts

Essential interventions and indicators (sources)	Bastar	Koraput	West Singhbhum
1. Improved food and nutrition intake			
% villages having PDS shops (HUNGaMA survey, 2011)	NA	45	81
% rural households using PDS service (HUNGaMA survey, 2011)	NA	42	61
2. Prevent micronutrient deficiency and anaemia			
% mothers consumed IFA tablets atleast 100 days (AHS 2012–13)	26.7	22.1	18.3
Malaria API (NVBDP 2012)	>10	>10	>10
% Plasmodium falciparum cases (DPMU, NHM (Bastar), Malaria Journal 2012^a^ (Koraput), DHFW 2014 (West Singhbhum)	95.2	89.1	80.3
3. Improving access to basic health and special care for at-risk			
%pregnant women registered in the first trimester (DLHS 3)	28	50	35
% mothers received at least one TT injection (AHS 2012–13)	88.7	96.8	86.7
% mothers receiving at least 3 ANC check-ups (AHS 2012–13)	69.4	74.9	61.4
% institutional delivery (AHS 2012–13)	67.1	53.4	38.5
% mothers receiving postnatal check-up(within 48 h) (AHS 2012–13)	77.2	67.5	53.9
4. Improving hygiene and sanitation and access to safe drinking water			
% households having access to hand pump or other safe drinking water systems (Census 2011)	77.7	73.9	58.6
% households having toilets (Census 2011)	20.3	17.4	11.8
% women reporting hand washing before preparing a meal (HUNGaMA survey, 2011)	NA	10	1
5. Preventing pregnancies too early, too many and too soon			
% women aged 20 to 24 who were married at 18 years or less (AHS 2012–13)	44.8	46.7	33.8
% women aged 20–24 reporting birth of order ≥3 (AHS 2012–13)	18.3	35.4	41.2
% use of modern contraceptive methods (AHS 2012–13)	48.2	33.6	32.2

*NA* (No PVTG in that district)

Coverage of second essential nutrition intervention on prevention of micronutrient deficiency and anaemia, was ascertained by assessing secondary data on consumption of iron folic acid (IFA) tablets for at least 100 days by pregnant women, consumption of iodized salt and incidence of parasitic infections like malaria and FGDs. Consumption of IFA tablets for at least 100 days by pregnant women was very low, ranging from 18.3% in West Singhbhum to 27% in Bastar. The most commonly cited reason for non-consumption of IFA tablets by pregnant women was unavailability of the tablets and early marriage, early pregnancy and poor diet intake for maternal anaemia. Annual Parasite Incidence or number of confirmed cases of malaria per 1000 population was greater than 10 in all study districts, which should be below 1 at state level to be categorised as low risk (Table 2).

The third essential nutrition intervention pertains to access to basic health care services and specialised care services for at-risk population, covering the entire period of pregnancy and post-partum. Only 50% of the pregnant women were registered within the first trimester in Koraput. The situation is worse in the other two districts. Despite delays in registration, tetanus toxoid (TT) vaccination services have nearly 100% reach in Koraput and close to 90% in other two districts. Koraput does better in reach of at least 3 antenatal check-ups with 75% of the pregnant women receiving this service, compared to about 70% in Bastar and over 60% in West Singhbhum. Institutional deliveries range from 38.5% in West Singhbhum to 67.1% in Bastar. Postnatal check-ups within 48 h of delivery are available to more women than those who delivered in an institution as service coverage ranges from 53.9 to 77.2% across study districts. Government’s financial assistance for institutional delivery has been utilised by over 90% of women who delivered in a health facility in the previous year in both Bastar and Koraput. Usage of this financial benefit is limited in West Singhbhum to 60% (AHS 2012–13, not included in Table 2).

Access to toilets is abysmally low, ranging from 12 to 20% across study districts; availability of drinking water ranges from 60 to 78%. Hand washing practices are inappropriate as almost all women in West Singhbhum and 90% in Koraput reported not washing hands before preparing a meal. Inappropriate hand washing practices are associated with increased risk of diarrhoea and respiratory infections [15]. Discussions revealed that girls are married young to ensure their security and settle dowry demands at the earliest. Nearly all women in Koraput and over 80% in West Singhbhum reported not having any decision-making power about household purchases. Decisions on family planning are also driven by men.

### Government programmes engaging community collectives

Various ministries have been promoting community collectives in the study districts (Table 3). The National Rural Livelihood Mission is the most recent initiative through which community collectives are being promoted, capacitated and monitored for both livelihood and social development. A federated structure is promoted through this mission involving SHG at tier 1, village organisations comprising 10 to 20 SHGs at tier 2 and block level Federations at tier 3. All mission-compliant SHGs are required to adhere to Panchsutras or five canons of democratic functioning, that is, (i) regular conduct of weekly meetings, (ii) member attendance at meetings, (iii) regular subscription of savings by members, (iv) inter-lending of SHG funds and (v) up-to-date bookkeeping. The management units at state, district and block level provide supervisory and capacity building support to SHGs and their federations. Table 4 provides positions filled and attrition rates of these management units [16].

**Table 3:** Ministries and linked programmes or initiatives through which community collectives are promoted in study districts

Ministry	Programmes/initiative	Bastar	Koraput	West Singhbhum
Ministry of Rural Development	NRLM Aajeevika	√	√	√
	Integrated Watershed Management Projects	√	√	√
Ministry of Tribal Affairs	ITDA	–	√^a^	–
Ministry of Women and Child Development	ICDS	√	√	√
	Mission Shakti	-	√	-
Ministry of Food and Civil Supplies	PDS	√	√	√
Ministry of Finance	NABARD, SHG-Bank linkage programme, Farmers clubs	√	√	√
Ministry of Health and Family Welfare	NRHM, Village Heath and Sanitation Committees	√	√	√
Ministry of Human Resource Development	Sarv Sikhsha Abhiyaan	√	√	√
Ministry of Environment, Forest and Climate Change	Forest conservation/Integrated Wildlife Management	√	√	√

√ implies Yes – implies not applicable

^a^Odisha Tribal Empowerment and Livelihood Project

**Table 4:** Positions filled and attrition rates^a^ at state, district and block management units of the SRLM

	Positions filled (%) and attrition rates (%)		
	State	District	Block
Chhattisgarh	79 (15)	55 (17)	77 (22)
Jharkhand	94 (4)	75 (7)	72 (4)
Odisha	43 (21)	21 (17)	60 (NA)

^a^Figure in parenthesis is attrition rate

*NA* (No PVTG in that district)

National Bank for Agriculture and Rural Development (NABARD) is the pioneer of SHG movement in the country, with the launch of its SHG-bank linkage programme dating back to 1982. It is actively involved in micro-credit activities in all three districts. The ITDA is the focal agency for holistic development of tribal population also promotes SHGs. The ITDA of Koraput is most evolved and implements Odisha Tribal Empowerment and Livelihood Programme as a holistic approach to tribal development including increasing access to land, water and forests, monitoring the basic food entitlements as well as promotion of local enterprise through SHGs and their federations. The state government of Odisha, through the Department of Women and Child Development has been promoting SHGs through its flagship programme—Mission Shakti since 2001which has a diversified capacity building plan, training members on business enterprise as well as socially relevant issues.

### Profile of community collectives

Based on the mapping of community collectives through 17, 28 and 33 promoting agencies in Bastar, Koraput and West Singhbhum, respectively, 18 different types of collectives exist. Six types of community collectives are common across study districts (Table 5). Amongst the six, SHGs are the only collective to have exclusive women membership and a federated structure (tier 2 and tier 3). It also has the maximum penetration with membership ranging from 68,332 to 205,171 across the three districts and all types of community collectives. In both Bastar and Koraput, government agencies are predominant promoters of SHGs and their federations—4708 of 6226 (76%) in Bastar and 14241 of the 18448 (77%) in Koraput are promoted by government. In West Singhbhum, government promoted SHGs form a smaller proportion at 44% (3200 of 7239).

**Table 5:** Common types of community collectives, their composition, structural tiers and membership across all study districts

S.No.	Type	Composition	Tiers			Number of members		
			I	II	III	Bastar	Koraput	West Singhbhum
1	Farmer Club	Mixed	√	X	X	4100	5086	4352
2	Forest Committee	Mixed	√	X	X	3520	16,517	2690
3	SHG	Women	√	√	√	68,332	205,171	117,558
4	Village Education Committee	Mixed	√	X	X	40,160	49,932	40,646
5	Village Health and Sanitation Committee^a^	Mixed	√	X	X	7080	17,282	–
6	Watershed Committees	Mixed	√	X	X	850	86,567	4750

√ implies yes, X implies no

^a^Referred to as Village Health, Sanitation and Nutrition Committees in Jharkhand

### Organisational and programme capacity of SHGs

**Fig. 3:**
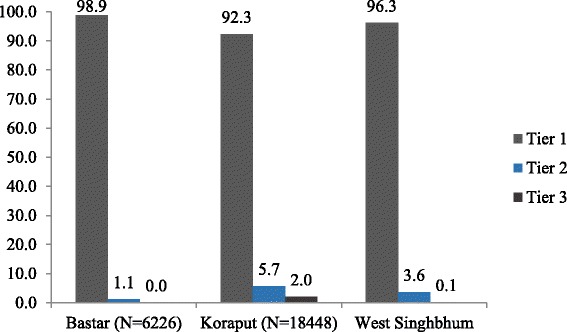
Distribution of SHGs based on the structural tiers

Most of the SHGs are yet to federate. The highest proportion of federated SHGs is in Koraput (Fig. 3). The proportion of tribal membership is 44% in Bastar, 51% in Koraput and 67% in West Singhbhum amongst selected “mature” SHGs. Proportion of members who can read and write is highest in West Singhbhum at 43%. Less than a quarter of the members can read and write in the other two districts. Leadership in SHG comprises the President, Secretary and Treasurer elected by the group members. With the exception of few SHGs, norms for election and/or rotation of leadership are not specified and members are not aware of the process in all three districts. The primary activity of all mapped SHGs across all study districts is thrift and credit which requires active bank accounts. Koraput has the maximum number of SHGs with active bank accounts (94%), followed by West Singhbhum (62%) and Bastar (41%). Regularity of savings and meetings was consistently high across study districts; most SHGs have financial penalties for absenteeism. The philosophy behind SHG development is to encourage business entrepreneurship at the village level. However, this is a weak area for most SHGs as very few are involved in group income generation activities. Typically, SHGs have been managing PDS, preparation of hot cooked meals for anganwadi centres, animal husbandry and fisheries. None of the selected SHGs had a vision or a long-term plan for increasing SHG operations and group income generation activities. The idea of savings is more individual need oriented rather than enterprise oriented. However, members did share a lot of aspirations and ideas, on activities that they could undertake as a collective. Some illustrative examples include poultry, pisciculture, spice packaging as well as management and/or monitoring of government schemes like PDS and ICDS.

These selected “mature” SHGs have no direct experience of working on women’s health and nutrition issues; neither did any members reported being trained on such themes in the past. FGDs revealed the need to improve diet during pregnancy through practical recommendations and awareness of benefits of services importantly, financial incentives for institutional deliveries and family planning.

On promotion of essential nutrition interventions, selected SHGs stated the possibility of (1) increasing food security by developing grain banks and homestead farming for nutritionally rich foods, (2) improving community and health centre linkages by liasoning with health and other service providers, (3) conducting social audits of ICDS and health services, (4) prioritising households with pregnant women and lactating mothers for construction of toilets and other development activities and (5) increasing awareness on women’s health and nutrition issues through the SHG meetings.

### Capacity of higher order (tier 2) community collectives

In this study, tier 2 collectives are all under the National Rural Livelihood Mission. The village organisation representatives are President and Secretary of the SHGs who are generally more vocal, active and literate members. Members meet monthly and a general body meeting of all SHGs members is organised every 2 months. The village organisations sanction loans up to Rs 60,000 (USD 873), referred to as Community Investment Fund at nominal interest rate, based on review of micro credit plans of the SHGs. This process creates an interface of SHGs and village organisations and sets accountability on either side.

Members are trained on micro credit planning, book keeping, organisation of general body meeting and leadership and provide monitoring and supervisory support to SHGs as a shared responsibility. Members are more networked and involved in rights-based advocacy with government agencies especially for access to piped drinking water supply, issuing ration cards and monitoring anganwadi centres. They are also more actively engaged in politics and administration.

### Perceptions of stakeholders in engaging community collectives as a stakeholder in promotion of essential nutrition interventions

Almost all stakeholders recognised that SHGs are a critical platform for reaching out to the women, assess vulnerability, prioritise households requiring assistance and reduce left-outs. SHG members are considered action-oriented, focused and reliable due to their disciplined and regular savings and meetings. However, village organisations are viewed as more powerful structures to undertake social and development causes; this also emerged from the capacity assessment.

Stakeholders identified barriers at three levels—SHGs themselves, the community and environment they operate in and the support structures available to them. At the SHG level, stakeholder reinforced that SHGs are not well-defined entities with respect to norms, maintenance of records, and they cannot challenge local political and administrative units. Their technical capacity for undertaking development work such as promotion of essential nutrition interventions was considered limited. Concerns were raised on (i) SHGs are not networked with frontline workers and service providers from other line departments; (ii) SHGs membership not being homogenous with respect to power and authority, that is, some SHG members may be influential and more controlling of the group; and (iii) need for continuous efforts for capacitating governance activities within the SHGs, while layering other health and nutrition promotion activities.

## Discussion

The research reveals high prevalence of chronic undernutrition and anaemia amongst women from three tribal-dominated districts of India, a finding that has been corroborated in other research as well [2, 17–19]. Despite relaxed population coverage norms for both nutrition and health service infrastructure for tribal areas, reach of essential nutrition interventions is limited in study districts where women are exposed to known socio-demographic and environmental determinants of chronic undernutrition. While both Jharkhand and Odisha have challenges in meeting health infrastructure norms for tribal areas, Chhattisgarh has a huge skilled human resource shortage [20].

In addition to the supply side barriers, focus group discussions revealed that care practices during pregnancy, perinatal and postnatal period were divergent from recommended due to limited information on appropriate practices, cultural beliefs and limited involvement of women in decision-making. Over 90% of mothers in Koraput and 88% mothers in West Singhbhum had never heard the term malnutrition [18]. Women need a collective force to voice their concerns and be an equal partner in the household; concomitantly men need to be sensitised to the need for joint decision-making, health and nutrition needs of women.

Amongst all collectives mapped, SHGs provide a platform for reaching the maximum number of like-minded, enterprising women, that is, nearly 400,000 in the three study districts. This coincides to approximately 20% of the total female population of about 2.2 million in these three districts, of which over 50% are from tribal communities. Evaluations of SHG programmes, with government or NGOs as promoting agencies of the SHG, have consistently reported improvement in economic status of members, level of improvement being contingent on original level of poverty and governance structures in the state or district [19, 20]. Evidence indicates that women from villages with SHGs are more likely to use health facilities for maternal and delivery care, are better informed and are more likely to use family planning methods than those from villages without SHGs [21]. Sampled SHGs in this study were cohesive, representative of the castes and tribes of the hamlet and met weekly providing a possible interface for discussing and resolving local challenges in implementation of essential nutrition interventions. The self-diagnosis of reasons for limited reach of essential nutrition services by SHGs is likely to improve targeting of interventions as they are most informed of local challenges.

Systems for establishing SHGs as partners in promotion and delivery of essential nutrition interventions are in place. In Koraput, government has a structured tribal welfare and development programme and has been involved in SHG promotion for a longer duration than in Bastar and West Singhbhum. Koraput has more than double the number of mapped SHGs compared to the other two districts. Sustained implementation of National Rural Livelihood Mission in convergence with ITDA can result in increasing the number of SHGs in these districts and reaching the mission’s target of enrolling at least one woman from all rural/tribal households in SHGs. However, research studies indicate that SHG membership alone, without other interventions, like supply strengthening, nutrition and health education amongst others, is not associated with better health and nutrition outcomes [22].

These SHGs from being a network of women involved in thrift and credit are ready to evolve into an organisation with defined operations in business enterprise and social development. This graduation is currently happening with SHGs federating to tier 2 organisations across the study districts. Long-term planning and sustainability of the SHGs should be the core intervention for embedding other interventions in this network. The mission, through its decade long plan of investing in local capacity development including identification and training of local resource persons, grading of SHGs based on compliance of the Panchsutras and promotion of a federated structure is strengthening the organisational structure and financial viability of the SHGs. However, the Mission’s management structures need to be strengthened in all three states. Lessons from the long standing SHG promotion schemes, namely, Mission Shakti and Odisha Tribal Empowerment and Livelihood Project should be brought forward to inform mission approaches.

## Conclusions

The study presents the unrelenting state of women’s undernutrition in the three tribal dominant districts of Bastar, Koraput and West Singhbhum. The current nutrition status of women is abysmally poor though not completely understood due to lack of data. In order to monitor and evaluate the effectiveness of the essential nutrition interventions in reducing undernutriton, including stunting, both women’s and children’s nutrition outcome indicators need to be included in large-scale district level surveys.

The presence of women collectives in these districts offers a potential solution to address the underlying cause of undernutrition that is, poverty, along with the empowering women as analysers, decision-makers and educators on essential nutrition interventions. Findings from this study suggest that, with capacity building and supervisory support, “matured” SHGs can develop plans and receive grants to strengthen last mile delivery of essential nutrition interventions. SHGs are at different levels of organisational evolution in the study districts. Through the National Rural Livelihood Mission, a grading of SHGs and their federations is available, which should be used to assess readiness of these groups to become grantees for promotion and delivery of essential nutrition interventions. Village organisations or tier 2 collectives should be equipped with technical know-how on essential nutrition interventions to model income generation activities linked community development through their vast network of SHGs. This scientifically developed, evaluated and documented model should be used to advocate for convergent action with other line departments and prioritising SHGs and their federations as the vehicle for promotion and delivery of essential nutrition interventions. Ministry of Health and Family Welfare, Ministry of Women and Child Development and Ministry of Rural Development are three potential ministries that should be targeted for engaging SHGs in improving food and nutrition security and access to health and sanitation facilities at village level. Ministry of Tribal Affairs should review the ITDAs and streamline the annual planning processes, operational strategies and monitoring mechanisms to establish ITDA as a convergence focal point for tribal development activities. In order to achieve full impact of SHG-led interventions, it is critical that concomitant efforts are made to improve reach of all public health and nutrition services. Study reveals gaps in reach of health and nutrition services but also indicates the better reach of TT vaccination and incentivized service such as financial assistance for institutional deliveries which should be reviewed to understand drivers for service utilisation. All national schemes that are aimed at universal coverage need to be reviewed with a tribal lens as improvements in national averages on women’s and children’s health and nutrition indicators are contingent on reaching out to the most vulnerable communities, that is the tribal and lowest economic quintile population.

## Abbreviations

AHS: Annual health survey
DLHS: District level household survey
FGDs: Focus group discussions
ICDS: Integrated child development services
IFA: Iron folic acid
ITDA: Integrated Tribal Development Agency
NABARD: National Bank for Agriculture and Rural Development
NGO: Non-government organisation
PDS: Public distribution system
SHGs: Self help groups
SRLM: State rural livelihood mission
TT: Tetanus toxoid
